# Tunable Schottky barrier in graphene/graphene-like germanium carbide van der Waals heterostructure

**DOI:** 10.1038/s41598-019-40877-z

**Published:** 2019-03-26

**Authors:** Sake Wang, Jyh-Pin Chou, Chongdan Ren, Hongyu Tian, Jin Yu, Changlong Sun, Yujing Xu, Minglei Sun

**Affiliations:** 1 College of Science, Jinling Institute of Technology, Nanjing, Jiangsu 211169 China; 2 Department of Mechanical Engineering, City University of Hong Kong, Kowloon Tong, Hong Kong 999077 China; 3 Department of Physics, Zunyi Normal College, Zunyi, Guizhou 563002 China; 4 School of Physics and Electronic Engineering, Linyi University, Linyi, Shandong 276005 China; 5 School of Materials Science and Engineering, Southeast University, Nanjing, Jiangsu 211189 China; 6 School of Materials Science and Engineering, Shandong University of Technology, Zibo, Shandong 255049 China; 7 Physical Science and Engineering Division (PSE), King Abdullah University of Science and Technology (KAUST), Thuwal, 23955-6900 Saudi Arabia

## Abstract

The structural and electronic properties of van der Waals (vdW) heterostructrue constructed by graphene and graphene-like germanium carbide were investigated by computations based on density functional theory with vdW correction. The results showed that the Dirac cone in graphene can be quite well-preserved in the vdW heterostructure. The graphene/graphene-like germanium carbide interface forms a p-type Schottky contact. The p-type Schottky barrier height decreases as the interlayer distance decreases and finally the contact transforms into a p-type Ohmic contact, suggesting that the Schottky barrier can be effectively tuned by changing the interlayer distance in the vdW heterostructure. In addition, it is also possible to modulate the Schottky barrier in the graphene/graphene-like germanium carbide vdW heterostructure by applying a perpendicular electric field. In particular, the positive electric field induces a p-type Ohmic contact, while the negative electric field results in the transition from a p-type to an n-type Schottky contact. Our results demonstrate that controlling the interlayer distance and applying a perpendicular electric field are two promising methods for tuning the electronic properties of the graphene/graphene-like germanium carbide vdW heterostructure, and they can yield dynamic switching among p-type Ohmic contact, p-type Schottky contact, and n-type Schottky contact in a single graphene-based nanoelectronics device.

## Introduction

Ever since Geim and Novoselov demonstrated the first isolation of graphene (G) in 2004^1^, two-dimensional (2D) material has been attracting much attention since its superior properties^2–6^ such as ultrahigh mobility of charge carriers at room temperature^7^, extreme mechanical strength^8^, superior thermal conductivities^9^, and high optical transmittance^10^. These properties render G very promising for catalysts^11–13^, nanoelectronic devices^6,14,15^, energy conversion and storage^16–20^, and sensors^21–24^. However, pristine G has a zero bandgap, which make it not suitable for many applications.

In recent years, G-like germanium carbide (GeC) have also attracted much interest. Unlike G, which is a semimetal, GeC is a direct bandgap semiconductor^25–28^. Its electronic properties are sensitive to the elastic strain^26,28^ and stacking effect^28,29^. For instance, Xu *et al*.^28^ found a semiconductor–metal transition can be induced by a biaxial tensile strain, while a direct–indirect bandgap transition triggered by a biaxial compressive strain. Multilayer GeC also exhibits a direct bandgap mimicking monolayer GeC, but its gap values decrease with the increase of the number of layers. Moreover, many studies show that the magnetic properties of GeC can be tuned by surface functionalization^30^, foreign atom adsorption^27^ and defects generation^31^. In addition, First principles calculations were also performed to understand the electronic and magnetic properties of GeC nanotubes^32–36^. Besides, the good stability of GeC monolayer has been demonstrated by the phonon dispersion^25^. All these investigations mentioned above suggest that GeC can be a vital 2D semiconducting material for many important applications.

Most recently, there has been rapidly growing interest in atomic-scale vertical van der Waals (vdW) heterostructures made from a combination of G and other 2D semiconducting materials, such as G/MoS_2_^37–40^, G/phosphorene^41–45^, G/arsenene^46,47^, G/blue phosphorene^48,49^, and G/g-GaN^50^. Such heterostructures preserve the unique Dirac cone structure of G and provide a higher electronic quality for G-based nanodevices. Moreover, they offer new opportunities for designing new electronic, optoelectronic, micromechanical, and other devices^51,52^. The abovementioned GeC monolayer provides a new platform for designing new G-based vertical vdW heterostructures, thus widening potential applications in nanodevices. We are unaware of any previous systematic studies on the electronic properties of layered G/GeC heterostructures, which are the focus of this investigation.

In this paper, we investigate the structural and electronic properties of the G/GeC heterostructure. The properties of both G and GeC were well-preserved upon their contact. Interestingly, by varying the interlayer distance, interlayer interactions in the G/GeC heterostructures could induce tunable Schottky barrier height (SBH). Moreover, the application of an external perpendicular electrical field also allows the control of SBH. These results can offer important information for the design of new devices based on G-based vdW heterostructures.

**Figure 1:**
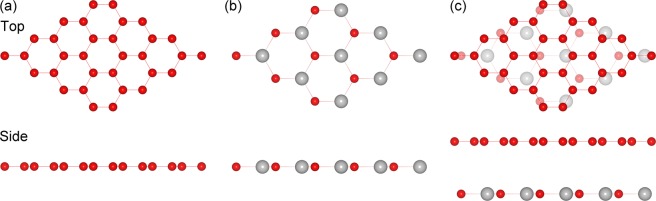
Schematic illustrations of the crystal structures of (**a**) G, (**b**) GeC, and (**c**) the G/GeC vdW heterostructures. The red and grey balls represent C and Ge atoms respectively.

To start with, we explored the geometric properties of pristine G and GeC monolayers. Figure 1(a) depicts the relaxed geometric structure of a G monolayer in a 4 × 4 supercell. The optimized lattice parameter of the G monolayer was 2.47 Å, which is in good agreement with the result of a previous study^53^. Figure 1(b) depicts the relaxed geometric structure of a GeC monolayer in a 3 × 3 supercell. The optimized lattice parameter of the GeC monolayer was 3.26 Å, which is also consistent with the values in previous reports^27,28,30,31^.

**Figure 2:**
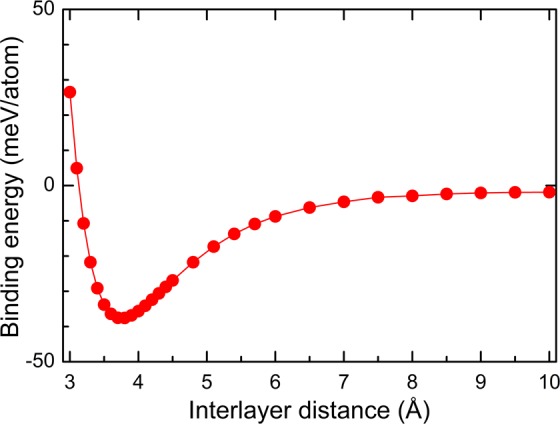
Binding energy of the G/GeC vdW heterostructures as a function of the interlayer distance (*D*).

Next, we designed a new 2D hybrid G/GeC heterostructure. To compensate for the lattice mismatch between G and GeC, we kept the GeC lattice fixed and compressed the G layer; the overall induced strain in the G lattice was only ~1.01%. We designed the G/GeC vdW heterostructures using a 4 × 4 G supercell and a 3 × 3 GeC supercell, and the equilibrium geometry of the G/GeC system is shown in Fig. 1(c). To quantitatively characterize the stability of the interface, we calculated the binding energy per atom of G, between G and GeC layers. The interface binding energy is defined as

1 $${E}_{{\rm{b}}}=[{E}_{{\rm{G}}/{\rm{GeC}}}-({E}_{{\rm{G}}}+{E}_{{\rm{GeC}}})]/{N}_{{\rm{G}}}$$

where *E*_b_ is the binding energy; *E*_G/Gec_, *E*_G_, and *E*_GeC_ are the total energy of the vdW heterostructures, compressed G, and pristine GeC respectively; and *N*_G_ is the number of carbon atoms in the G layer of the heterostructure system. Based on this equation, the binding energy is given in Fig. 2 as a function of interlayer distance (*D*) between G and GeC. One observes that when the interlayer distance was about 3.75 Å, the corresponding binding energy was lowest. Based on the equilibrium position of the GeC layer with respect to the G layer, a small binding energy of about −38 meV/atom was obtained. This binding energy is the same as that in other vdW nanostructures such as graphite and bilayer hexagonal boron nitride^54^. Thus, the weak vdW interactions played a dominant role in G/GeC system, in which the superb electronic structures of G will be well persevered.

**Figure 3:**
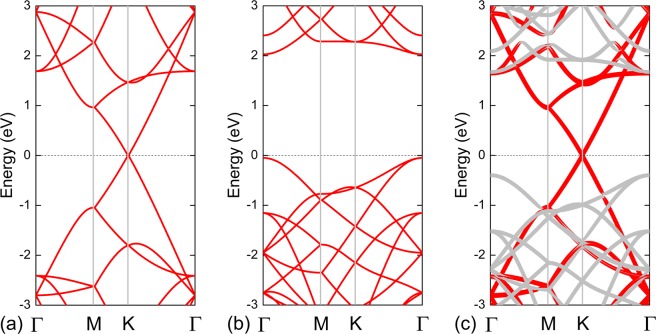
Band structures of (**a**) 4 × 4 G and (**b**) 3 × 3 GeC systems and projected band structures of (**c**) G/GeC vdW heterostructures with an interlayer distance of 3.75 Å. The red and grey symbols represent G and GeC, respectively. The zero-energy value corresponds to the Fermi level.

We then continued to explore the electronic properties of the G/GeC vdW heterostructures. The electronic properties of pristine G and GeC in their origin scheme were checked first, and their electronic band structures are shown in Fig. 3(a,b). It is clear that G was a semimetal, exhibiting a linear Dirac-like dispersion relation around the Fermi level (Fig. 3(a)). The GeC monolayer was semiconducting with a direct bandgap of 2.08 eV. This value is in good agreement with previous theoretical studies^25,27,31^ even use different code and functional. The conduction band minimum (CBM) and valence band maximum (VBM) of the GeC monolayer are both located at Γ point, as shown in Fig. 3(b). Fig. 3(c) shows the projected band structure of the G/GeC vdW heterostructure in its most stable configuration; the contribution of the carbon atoms in G is in red, and the contribution of the germanium and carbon atoms in the GeC monolayer is in grey. The G part retained its semimetallic behaviour. Compared to freestanding G, the Fermi velocity at the Dirac cone was almost unchanged in the G/GeC vdW heterostructure, and no bandgap was opened at the Dirac cone of G. Meanwhile, the GeC part of the G/GeC vdW heterostructure retained its semiconducting characteristics and had a bandgap of 2.07 eV. Compared with the bandgap of the separate GeC monolayer (Fig. 3(b)), the decrease in the bandgap (0.01 eV) may be originated from the weak interfacial interaction between the π cloud of G and the p_*z*_ orbitals of GeC. Consequently, the excellent intrinsic electronic properties of both G and GeC layers were found to be quite well-conserved upon binding.

**Figure 4:**
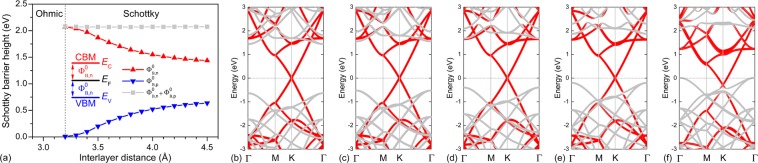
(**a**) Schottky barriers ${{\rm{\Phi }}}_{{\rm{B}},{\rm{n}}}^{0}$, ${{\rm{\Phi }}}_{{\rm{B}},{\rm{p}}}^{0}$, and ${{\rm{\Phi }}}_{{\rm{B}},{\rm{n}}}^{0}$ + ${{\rm{\Phi }}}_{{\rm{B}},{\rm{p}}}^{0}$ in G/GeC vdW heterostructures as functions of the interlayer distance. Band structures of G/GeC vdW heterostructures with different interlayer distances of (**b**) 4.5 Å, (**c**) 4.0 Å, (**d**) 3.75 Å, (**e**) 3.5 Å, and (**f**) 3.0 Å. The zero-energy value corresponds to the Fermi level.

From Fig. 3(c), we also discovered that a Schottky contact formed at the G/GeC interface, just like coupling G with phosphorene^41–44^, arsenene^46,47^, blue phosphorene^47,49^, and g-GaN^50^ in previous works. The fact that no gap states are formed within the bandgap of GeC denotes that Fermi level pinning is absent in G/GeC Schottky contact. Therefore, the SBH in this contact can be directly determined by Schottky–Mott rule^45,46^. Based on the Schottky–Mott rule^45,46^, the corresponding SBH was determined by the energy levels of band edges in the semiconductor and the Fermi level in the metal^55^. Therefore, the n-type SBH (${{\rm{\Phi }}}_{{\rm{B}},{\rm{n}}}^{0}$) is the energy difference between the CBM of the GeC and the Dirac cone of the G, while the p*-*type SBH (${{\rm{\Phi }}}_{{\rm{B}},{\rm{p}}}^{0}$) is the energy difference between the Dirac cone of the G and VBM of the GeC. Furthermore, the sum of two types of Schottky barrier was roughly equal to the bandgap (*E*_g_) of the semiconductor, that is, ${{\rm{\Phi }}}_{{\rm{B}},{\rm{n}}}^{0}+{{\rm{\Phi }}}_{{\rm{B}},{\rm{p}}}^{0}\approx {E}_{{\rm{g}}}$. The Schottky barriers ${{\rm{\Phi }}}_{{\rm{B}},{\rm{n}}}^{0}$, ${{\rm{\Phi }}}_{{\rm{B}},{\rm{p}}}^{0}$, and ${{\rm{\Phi }}}_{{\rm{B}},{\rm{n}}}^{0}$ + ${{\rm{\Phi }}}_{{\rm{B}},{\rm{p}}}^{0}$ in the G/GeC vdW heterostructures are shown in Fig. 4(a) as functions of the interlayer distance. In general, the interface forms a p-type Schottky contact when *D* is larger than 3.2 Å. In these systems, conduction occurred through holes. When *D* = 4.5 Å, a p-type SBH of 0.64 eV was obtained (Fig. 4(b)). As the interlayer distance was decreased from 4.5 to 3.0 Å, the position of the Dirac cone moves close to VBM of the GeC monolayer (Fig. 4(b–f)). In the G/GeC vdW heterostructure with *D* = 3.75 Å, the ${{\rm{\Phi }}}_{{\rm{B}},{\rm{p}}}^{0}$ was 0.40 eV, which is much smaller than ${{\rm{\Phi }}}_{{\rm{B}},{\rm{n}}}^{0}$ of 1.67 eV (Fig. 4(d)). Therefore, the G/GeC vdW heterostructure at the equilibrium distance is a p-type Schottky contact. When *D* was decreased to 3.2 Å, the Fermi level of the system will intersect the VBM of GeC layer, indicating a p-type Ohmic contact (Fig. 4(a)). Upon further decrease of the interlayer distance from 3.2 to 3.0 Å, the system was still p-type Ohmic (Fig. 4(f)).

**Figure 5:**
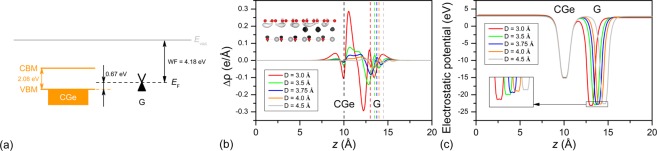
Plots of (**a**) energy level disposition for the compressive G and GeC monolayer. *xy*-averaged (**b**) differential charge density and (**c**) electrostatic potential of G/GeC vdW heterostructures with different interlayer distances of 3.0, 3.25, 3.5, 4.0, and 4.5 Å in the *z* direction. The isosurface of charge difference at the equilibrium distance (*D* = 3.75 Å) is shown in the inset of (**b**). The black and white regions denote the gain and loss of electrons, respectively. The depths of potential wells of G are shown in the inset of (**c**).

To understand the underlying mechanism for transition of the p-type Schottky contact to Ohmic contact in the G/GeC vdW heterostructures, we calculated the band alignment of G and GeC layer, *xy*-averaged differential charge density and electrostatic potentials at different values of *D* in the *z* direction, as shown in Fig. 5. For a compressive G layer (1.01%), the work function (WF) is 4.18 eV. It is clear that the Dirac cone of G was closer to the VBM of GeC (Fig. 5(a)). Therefore, the G and GeC interface will form a p-type Schottky contact for a nearly infinite value of *D* (such as *D* = 4.5 Å). As the interfacial distance was decreased from 4.5 to 3.0 Å, the effects of interlayer interactions and charge transfer between G and GeC were strengthened (see Fig. 5(b)). Bader analysis^56–58^ also demonstrated that when *D* was decreased from 4.5 to 3.0 Å, more electrons (0.0286, 0.03830, 0.04290, 0.0471, and 0.1042 |e| for *D* = 4.5, 4.0, 3.75, 3.5 and 3.0 Å, respectively) transferred from G to GeC, shifting down the energy level of G close to the VBM of GeC, as shown in Fig. 5(c), and finally inducing a p-type Ohmic contact.

**Figure 6:**
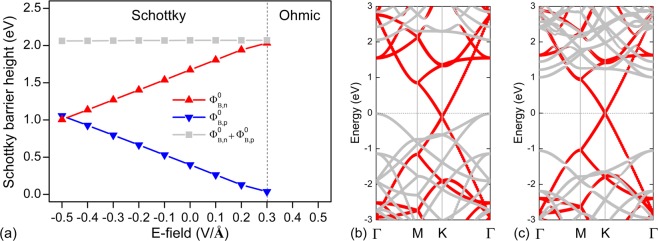
(**a**) Schottky barriers ${{\rm{\Phi }}}_{{\rm{B}},{\rm{n}}}^{0}$, ${{\rm{\Phi }}}_{{\rm{B}},{\rm{p}}}^{0}$, and ${{\rm{\Phi }}}_{{\rm{B}},{\rm{n}}}^{0}$ + ${{\rm{\Phi }}}_{{\rm{B}},{\rm{p}}}^{0}$ in G/GeC vdW heterostructures as functions of the E-field. Band structures of G/GeC vdW heterostructures under an E-field strength of (**b**) *E* = 0.4 V/Å and (**c**) *E* = −0.5 V/Å. The Fermi level of the systems was set to zero.

The application of a perpendicular electric field (E-field) has proved to be a rather effective way to tune the electronic properties of 2D materials^59–64^. Very recently, Padilha *et al*.^41^ demonstrated that by applying a perpendicular E-field, it was possible to control the SBH of heterostructures constructed by combining monolayer and bilayer phosphorene with G. Encouraged by this investigation, we also explored the effect of an external E-field on the electronic properties of the most stable G/GeC vdW heterostructure in our study (with *D* = 3.75 Å). The Schottky barriers ${{\rm{\Phi }}}_{{\rm{B}},{\rm{n}}}^{0}$, ${{\rm{\Phi }}}_{{\rm{B}},{\rm{p}}}^{0}$, and ${{\rm{\Phi }}}_{{\rm{B}},{\rm{n}}}^{0}$ + ${{\rm{\Phi }}}_{{\rm{B}},{\rm{p}}}^{0}$ in the G/GeC vdW heterostructure are shown in Fig. 6(a) as functions of the E-field. The E-field pointing from the G monolayer to the GeC substrate was defined as the positive direction. As can be seen in Fig. 6(a), applying a positive E-field shifted the Dirac cone of G closer to the valence band of GeC, and it will finally induce the transition from a p-type Schottky to a p-type Ohmic contact when *E* = + 0.4 V/Å (Fig. 6(b)). When a larger positive E-field was applied, the system would remain as a p-type Ohmic contact with further increases in the E-field strength (Fig. 6(a)). In contrast, for a negative field, the Dirac cone moved closer toward the conduction band, resulting in the transition from a p-type Schottky contact to an n-type Schottky contact when *E* decreased to −0.5 V/Å (Fig. 6(c)). Thus, it was able to achieve dynamic switching between n-type Schottky, p-type Schottky, and p-type Ohmic contacts in one heterostructure by applying an E-field, which is very useful for the design of novel Schottky devices such as a Schottky barrier transistor with high on/off current ratio. One can estimate the on/off current ratio in a Schottky device based on the diode equation^65^:

$${T}^{2}\text{exp}\frac{-q{{\rm{\Phi }}}_{\text{B}}^{0}}{{k}_{\text{B}}T},$$

where the *T, q*, ${{\rm{\Phi }}}_{{\rm{B}}}^{0}$, and *k*_B_ represent the temperature, the elementary charge, the SBH, and the Boltzmann constant respectively. We estimate that the on/off current ratio in a G/GeC Schottky contact based transistor at room temperature can reach as much as 10^7^.

In summary, the structures and electronic properties of G/GeC vdW heterostructure were investigated by density functional theory computations with vdW correction. The results demonstrated that the electronic properties of G/GeC vdW heterostructure were well-preserved upon their contact. Moreover, the p-type Schottky barrier height can be effectively modulated by varying the interlayer distance: it decreased as the interlayer distance decreased from 4.5 to 3.0 Å and finally transformed into a p-type Ohmic contact. In addition, the Schottky barrier height can also be tuned by application of an external E-field: the positive electric field resulted in a p-type Ohmic contact, while the negative electric field remarkably induced the transition from a p-type Schottky contact to an n-type Schottky contact. All of these excellent properties are essential for the application of G-based vdW heterostructures in novel nanodevices.

## Methods

First-principles calculations were performed by using the Vienna *ab initio* simulation package^66–69^, which uses a plane-wave basis set and projector-augmented wave pseudopotentials^70^ with Perdew–Burke–Ernzerhof^71,72^ exchange and correlation functional. A plane wave basis set with an energy cutoff of 550 eV was used in this study. The Brillouin zone integration was sampled in ***k****-*space within a Γ-centred scheme by 10 × 10 × 1 mesh points. The energy and force convergence were 10^−6^ eV and 0.01 eV/Å respectively. In order to accurately describe the long-range interactions, vdW correction proposed by Grimme (DFT-D3)^73^ was selected. A large vacuum space of 20 Å was adopted to eliminate interactions between the neighbouring slabs. Dipole correction was applied in all the calculations.
